# Effect of Dexmedetomidine Added to Lidocaine Cartridge on the Level of Patient Sedation, Cooperation, and Patient and Surgeon Satisfaction during Mandibular Third-Molar Extraction Surgery: A Randomized Double-Blind Controlled Trial

**DOI:** 10.1155/2022/4722674

**Published:** 2022-09-23

**Authors:** Milad Etemadi Sh, Nasser Kaviani, Kimia Salimian, Golnaz Tajmiri

**Affiliations:** ^1^Department of Oral and Maxillofacial Surgery, Dental Implants Research Center, Dental Research Institute, School of Dentistry, Isfahan University of Medical Sciences, Isfahan, Iran; ^2^Department of Oral and Maxillofacial Surgery, Dental Research Center, Dental Research Institute, School of Dentistry, Isfahan University of Medical Sciences, Isfahan, Iran; ^3^Dental Students' Research Committee, School of Dentistry, Isfahan University of Medical Sciences, Isfahan, Iran; ^4^Dental Implants Research Center, Dental Research Institute, School of Dentistry, Isfahan University of Medical Sciences, Isfahan, Iran

## Abstract

**Background:**

Various methods have been introduced for anxiety control during third-molar extraction surgery. Dexmedetomidine (DEX) is known to have analgesic, anxiolytic, and sympatholytic properties with minimal adverse effects. This study aimed to evaluate the impact of the local injection of the combination of DEX and Lidocaine on patients' anxiety and the surgeon's satisfaction during third-molar extraction surgery.

**Methods:**

A total number of 26 healthy volunteers with symmetrical bilateral impacted mandibular third-molar teeth indicated for surgical removal were included in this double-blind randomized controlled trial. A single experienced surgeon performed two surgical extraction procedures within at least four-week time intervals using anesthetic cartridges containing “DEX + LIDO” or “LIDO alone” used randomly on each side for each patient. The Visual Analog Scale and the SDFQ index were used to evaluate patients' anxiety and surgeon satisfaction during the procedure.

**Results:**

SDFQ reports showed that patients in the “DEX” group were 1.5 times more relaxed than those in the “LIDO alone” group. As a result, the level of sedation was considered statistically significant between the two groups (Wilcoxon test, *P* value <0.019). Wilcoxon test results also showed significant differences between the two groups regarding patients' overall cooperation in terms of interfering movement and verbal presentation of discomfort (*P* value <0.05); however, this difference was not considered significant regarding nonverbal signs of discomfort (*P* value >0.05). Moreover, both the surgeon and the patients reported a significantly higher satisfaction rate in the DEX group (paired *T*-test, df = 25, *P* value <0.05).

**Conclusions:**

It was inferred from the outcomes of the present study that the application of DEX added to the LIDO local anesthesia cartridge could significantly benefit anxious patients with previous unpleasant dental treatment experiences. *Trial Registration.* This trial is registered with the clinical trial registration number: IRCT20200406046966N.

## 1. Introduction

Many patients experience anxiety before, during, and after surgical dental treatments due to the expected pain and discomfort. Experience of anxiety and discomfort following dental treatments, including impacted third-molar extraction surgery as the most common dental surgical procedure [1], may lead to an irrational fear of other necessary dental treatments [2]. On the other hand, the role of dental anxiety in a patient's perception of pain, cooperation, and satisfaction cannot be ignored [3]. Patients with a high level of anxiety who agree to be operated on under local anesthesia would eventually be unable to cooperate adequately during the procedure, which results in stress on the operator, poor surgical performance, and a prolonged operating time [4]. Also, anxious patients tend to exaggerate the intensity of their pain and fear [5].

Besides, the application of sedation methods in complex rehabilitations as well as certain conditions including temporomandibular joint disorders, bruxism, and masticatory muscle spasms enhances patients' cooperation and the quality of treatment [6, 7]. Numerous medications and methods have been discovered, used, and studied over the years for managing dental fear and anxiety [8]. Midazolam is a well-known benzodiazepine commonly administered by the intravascular (IV) route during oral surgery for sedative purposes [9]. Since it can cause some respiratory depression alongside the absence of analgesic properties, some investigators are looking for an alternative with an easy and effective route of administration as well as additional analgesic properties and minimal adverse effects.

Achieving profound local anesthesia plays a critical role in the success of a surgical procedure [10]. Various adjunctive agents are added to local anesthetics to improve analgesic efficiency and reduce the required number of anesthetic cartridges [11]. DEX (Table 1) is a colorless medication that acts as a selective alpha-2 adrenoreceptor agonist, acting in various tissues, including the nervous, cardiovascular, and respiratory systems [12]. It was approved by the FDA in 1990 for sedation of non-intubated patients during surgical procedures [13]. When used as a sedative, DEX is believed to have analgesic, anxiolytic, and sympatholytic properties along with minimum respiratory and neurocognitive adverse effects [14, 15]. It is also associated with reduced blood pressure and heart rate [16]. DEX also has vasoconstrictor features by acting on alpha-2 *β* receptors of peripheral blood vessels and has been shown to enhance the anesthetic effects and prolong the duration of action of LIDO (Table 1) local anesthesia as well as reduce the required dosage of anesthetic drugs [17]. Therefore, it is suggested that DEX can be helpful for anxiety control in patients undergoing dental surgery when achieving adequate patient cooperation is a challenging issue for the operator [18].

It was demonstrated that during the recovery period, the analgesic and sedative effects of DEX were longer than the combination of midazolam and fentanyl [19]. Also, DEX combined with bupivacaine, compared to bupivacaine alone, provides a better anesthetic effect due to the prolonged analgesic effect in greater palatine injection during cleft palate surgery. Moreover, the severity of the pain in the first 24 hours was reported to be less by using this combination, while sedative and hemodynamic properties were similar in both groups [20]. Furthermore, adding a small amount of DEX to LIDO and its application for nerve block injection has been demonstrated to decrease the onset time of anesthesia and increase the duration of action without causing any systemic side effects [21, 22].

Another study by Nooh et al. showed that 1.5 *µ*gr/kg inhaling DEX could significantly increase patient calmness in the first 20–30 minutes of the surgical procedure; the peak effect was in 40–50 minutes after application and eliminated after 70–80 minutes. Besides, systolic pressure and heart rate were lower in this group. The conclusion was that DEX could be considered a safe and efficient sedative for the surgical removal of impacted third-molar teeth [23].

The results of a study by Fan and colleagues showed lower blood pressure and heart rate during dental surgical procedures in patients receiving DEX; furthermore, a higher cooperation rate was reported in the DEX group in comparison to the midazolam group. No respiratory adverse effects were reported for the DEX group. Therefore, regarding predictable hemodynamic and pharmacologic properties, DEX is suggested to be a safe substitute for midazolam for sedation purposes [24].

On the contrary, Cheung and associates showed that the sedative effects of DEX (0.88 *µ*gr) were comparable to midazolam (3.6 *µ*gr), while blood pressure, heart rate, and anxiety in the DEX group were considerably lower than midazolam. The patient's satisfaction in the midazolam group was not superior to the DEX group [25]. Moreover, in a systematic review, it was reported that there was no statistical difference between the two routes of intraoral submucosal and extraoral intramuscular injections of DEX regarding postoperative pain, swelling, and trismus [26].

The present study aimed to evaluate the efficacy of DEX as an adjunctive agent to LIDO in reducing patients' anxiety and improving cooperation during surgery.

## 2. Materials and Methods

Calculation of the sample size was done using the formula for comparison of two means. A minimum difference of 1.3 in the mean score of the Visual Analog Scale (VAS) (Table 1) between the “LIDO alone” and “LIDO + DEX” groups was considered statistically significant with an *α*-error of 0.05 and 1-*β* = 0.80. This randomized double-blind controlled trial study with a split-mouth and parallel design was performed on 26 patients (52 samples) with bilateral impacted mandibular third molars indicated for surgical removal who were referred to the Especial Dental Clinics of Isfahan University of Medical Sciences, Isfahan, Iran, from April to July 2020. None of the patients or the surgeon were aware of the type of anesthetic agent used for each surgery.

This study followed the Declaration of Helsinki on medical protocol and ethics, and the Regional Ethical Review Board of Isfahan University of Medical Sciences approved this study on 4th April 2020 (IRB approval code: IR.MUI.RESEARCH.REC.1399.009). Before the intervention, informed consent was obtained from all participants. The unwillingness of the patient to continue participation in the study came into consideration. All patients' information was kept confidential in this study. This study was registered in the Iranian clinical trial registry IRCT20200406046966N.

Healthy participants (ASA class I or II) of both sexes aged between 18 and 30 with asymptomatic bilateral mesioangular Class I and II position B impacted mandibular third molars indicated for surgical removal were included in the present study. To ensure that the nature of impaction was the same on both sides, radiographic evaluation using a panoramic view was done, and too easy or too challenging cases were excluded from the study. Having low blood pressure, bradycardia, unstable hypertension, and liver disorders that are contraindications for the application of DEX also came into consideration. The presence of tumors and malignant lesions and being a smoker were considered exclusion criteria. Being allergic to DEX and/or LIDO, surgical duration of more than 20 minutes, application of more than one cartridge, patients who did not come back for the second surgery, and the occurrence of medical complications during surgery were among other exclusion criteria.

Patients were allowed to rest for 10–15 minutes before starting the surgery. Considering the split-mouth and crossover study designs in all participants, patients were randomly divided into two subgroups using a computer-generated random list. In group 1 (*n* = 13), the first surgery was performed under local anesthesia only (cartridge containing 1.8 mL of 2% LIDO with epinephrine 1 : 80,000), and in group 2 (*n* = 13), the first surgery was performed by applying DEX in combination with local anesthesia (cartridge containing 1.8 mL of 2% LIDO with epinephrine 1 : 80,000 + 0.1 ml DEX [27]), and for the second surgery, the subjects received the anesthetic solutions in the reverse order.

As a result, since DEX has a different pH compared to LIDO local anesthetic, the pH of the local anesthetic would most likely change; as a result, it is essential to keep the preparation's consistency under control [28]. 0.1 ml of DEX (Daru Pakhsh Co., Tehran, Iran), which contains ten µg of the drug, was added to each LIDO cartridge (Exir Co., Tehran, Iran) using an insulin syringe. To equalize the volume and appearance of cartridges, 0.1 ml of normal saline was added to cartridges in the control group. Both solutions were prepared by an anesthesiologist with matching random codes registered on them. The observers and the attending surgeon were blind regarding the type of cartridge being used for each surgery.

The surgical procedure started 15 minutes following the local anesthetic injection. The surgeries were carried out in a fully equipped operating room with resuscitation equipment. Standardized surgical technique was applied by providing an envelope flap followed by a buccal osteotomy, tooth removal, copious rinsing, suturing, and patient instruction. The duration of surgery was recorded as the period between making the incision and placing the last stitch.

Patients did not take any medication before surgery. Two surgeries were performed by one experienced surgeon, separated by at least four weeks.

**Figure 1:**
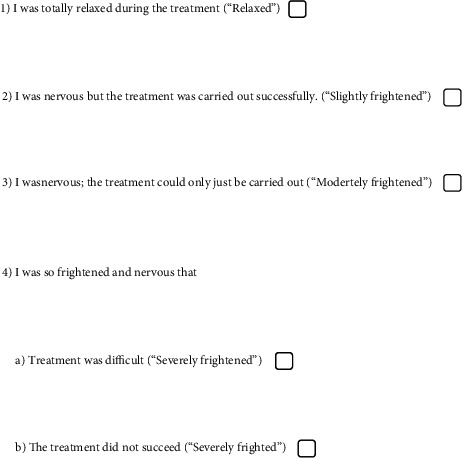
The SDFQ and clinical classification of patient's fear.

At the end of the surgery, patients were asked whether they were relaxed or anxious during the operation using a short dental fear questionnaire (SDFQ) as a 4-point Likert scale that shows the greater numerical value for greater fear (Figure 1) [29]. Patients' cooperation was assessed by a cooperation scale consisting of three parts (Table 2) [30], and their overall satisfaction was evaluated by a linear, nongraded 10 cm VAS assessed by a blind observer at the end of the procedure [31]. VAS scores of satisfaction of both patient and surgeon were recorded between 0 (not satisfied at all) to 10 (very satisfied) [32]. After the second procedure, the patients were asked to subjectively evaluate the two procedures in terms of anxiety and discomfort and to indicate which application was more effective or if they were equally effective in reducing their anxiety.

Statistical analysis was performed by setting the significance level at 0.05 and using SPSS (Table 1), version 22. Patients' sedation, cooperation, and patient's and surgeon's satisfaction scores were compared using the Wilcoxon and paired *T* test.

## 3. Results

Of the total number of 26 patients, 58% were female. The mean age of the participants was 22.03 ± 2.39.

SDFQ reports showed that patients in the “DEX + LIDO” group were 1.5 times more relaxed than those in the “LIDO alone” group, which was considered statistically significant (Wilcoxon test, *P* value <0.019) (Table 3).

**Figure 2:**
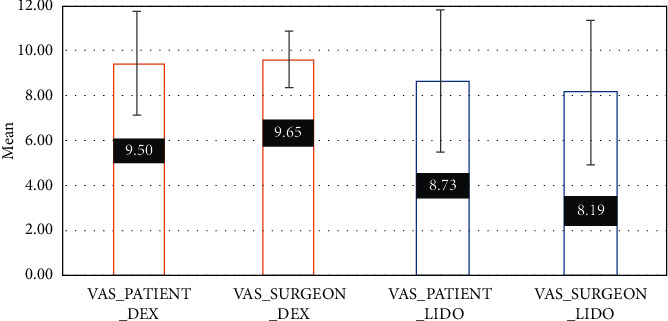
VAS scores of the patients and the surgeon.

The mean scores of satisfaction for both the surgeon and the patients were significantly higher in the DEX + LIDO group (Figure 2) (paired *T* test, df = 25, *P* value <0.05); however, differences between patients' and surgeon's satisfaction were not significant in each DEX and LIDO group (paired *T* test, df = 25, *P* value <0.05) (Table 4).

Regarding patients' cooperation, there was a significant difference between the two groups concerning interfering movement and verbal presentation of discomfort (Wilcoxon test, *P* values of 0.021 and 0.040, respectively). Nonetheless, nonverbal signs of discomfort did not differ significantly between the DEX + LIDO group and the LIDO alone group (Wilcoxon test, *P* value = 0.050) (Tables 2 and 5).

## 4. Discussion

The results of the present study showed that the surgeon's and patient's satisfaction was significantly higher by adding DEX to the LIDO cartridge when compared to LIDO alone. Besides, the patient's level of sedation and cooperation was shown to be significantly higher in the DEX group.

Concerning the degree of sedation, a systematic review and meta-analysis have reported that DEX as a safe medication can provide a good sedative effect for surgical extraction of impacted wisdom teeth [27]. These results are in favor of the outcomes of our study.

On the contrary, several studies have evaluated the effect of DEX on patients' sedation. According to Obayah et al. during the first 24 hours after surgery, no significant change in the sedation level was reported among children who received greater palatine nerve blocks using bupivacaine plus DEX and those who received bupivacaine alone [20]. Furthermore, Cheung et al. compared DEX to midazolam for sedation purposes in third-molar extraction surgery and discovered that both medications provided comparable sedation. Heart rate and blood pressure were reported to be lower in the DEX group. There was no significant difference in satisfaction or pain scores between the two groups [18]. These contradictory results could be explained by the different control groups studied in these experiments compared to our study. In fact, DEX is compared to midazolam as a sedative or bupivacaine as long-lasting local anesthesia was shown to be effective in pre and postoperative pain control, which can directly affect patient satisfaction when compared to LIDO as a medium-lasting agent with no considerable sedative properties.

Regarding pain evaluation, Gursoytrak et al. showed that submucosal administration of DEX added to articaine local anesthesia could be considered an effective tool for controlling postoperative symptoms including edema, trismus, and pain [29]. However, when comparing DEX to ketamine in another study with a similar design, it was demonstrated that ketamine was superior to DEX concerning postoperative pain control [31]. The outcome of our study supports the results of their first trial and the efficacy of DEX in postoperative pain control. Moreover, contradictory results with the second experiment could be justified by the fact that DEX is compared to ketamine, which as an opioid is considered the most potent medication available for pain control and is not used routinely for this purpose following surgical removal of impacted mandibular third-molar teeth. Besides, other studies have stated that LIDO plus DEX compared to LIDO alone could reduce the pain score significantly. In Cheung's study, evaluating the effects of intranasal administration of DEX 45 minutes before third-molar extraction surgery as an adjunct to the local anesthesia, perioperative sedation, and postsurgical analgesic effects was reported to be superior in the DEX group [32]. Also, Nooh et al. reported that intranasal administration of 1.5 *μ*g/kg atomized DEX is effective, convenient, and safe as a sedative for patients undergoing third-molar extraction [23]. Besides, DEX also provided satisfactory premedication sedation when used alone intranasally in children [33]. Also, according to Tonooka and colleagues, administration of DEX added to LIDO for dental local anesthesia in eight patients led to improved sedation levels and enhanced pulpal local anesthesia compared to LIDO alone [34]. Similar results were obtained from other clinical studies by Alizargar, Sing, and colleagues [35, 36]. These results are consistent with the outcomes of our study and support the idea that DEX, regardless of the route of administration, could provide superior results concerning the level of sedation and patients' cooperation.

During a dental surgical procedure, the bispectral index tracked a comparison of conscious sedation using DEX and Midazolam; findings showed that patients in the DEX group had lower heart rates and blood pressure and cooperated better. However, there was no noticeable difference in bispectral index values between the two groups [24]. Furthermore, in Ustun's study, it was reported that although both groups experienced equal levels of sedation, the cooperation scores revealed that the operator preferred DEX. In conclusion, these results suggest that DEX can provide a higher level of satisfaction for both the operator and the patient when compared to midazolam [37]. These results were consistent with our work. Regarding the patients' cooperation, however, a thorough evaluation of different aspects of cooperation in our study, including interfering movement and verbal and nonverbal representation of discomfort showed that application of DEX led to significant improvement in the first two mentioned factors, while the nonverbal representation of discomfort was shown to be comparable between the two groups. This can be justified by the fact that nonverbal representation of discomfort in contrast to interfering movements and verbal complaints during surgery might not directly interfere with the surgeon's access and vision to the surgical site and, as a result, could be more easily ignored.

Regarding the split-mouth design of the study, more reliable outcomes were achieved owing to the controlled confounding factors. Nevertheless, this study had some limitations. Increasing the dosage of DEX, in addition to the sample size, can lead to more comprehensive evaluations, alongside investigating other confounding factors. Selecting samples from a wider age range might help control other confounding factors.

It could be inferred from the results of the present study that in patients with a high level of anxiety and/or unpleasant previous experience, DEX added to LIDO local anesthetic is suggested to reduce stress level as well as improve patients' cooperation. We suggest that patients' preoperative stress levels would be measured and recorded using a questionnaire, and only patients with high-stress levels should be included in future studies so that the effect of this method on truly anxious patients can be measured.

## Figures and Tables

**Table 1:** Table of abbreviations for common terms.

	Term	Abbreviation
1	DEX	DEX
2	Visual analog scale	VAS
3	Short dental fear questionnaire	SDFQ
4	Lidocaine	LIDO
5	Food and drug administration	FDA
6	Statistical package for social sciences	SPSS

**Table 2:** Patients cooperation values were reported in three parts including interfering movement, verbalized discomfort, and nonverbal signs of discomfort.

Patients cooperation			Local anesthetic cartridge		Total
			Dex + lido	Lido alone	
Interfering movements	No interfering movement	Number	24	16	40
		Percent	92.3%	61.5%	76.9%
	Minor movements, the position remained appropriate	Number	2	10	12
		Percent	7.7%	38.5%	23.1%
	Minor movements, the patient had to be repositioned	Number	0	0	0
		Percent	0	0%	0%
	Gross movements considerably interfered with the procedure	Number	0	0	0
		Percent	0%	0%	0%

Verbalized discomfort	Not at all	Number	20	12	32
		Percent	76.9%	46.2%	61.5%
	Some verbalization but did not indicate pain or discomfort	Number	3	8	11
		Percent	11.5%	30.8%	11.5%
	Some verbalization indicating pain or discomfort	Number	3	6	9
		Percent	11.5%	23.1%	17.3%
	Complained frequently during the procedure	Number	0	0	0
		Percent	0%	0%	0%

Nonverbal signs of discomfort	Not at all	Number	21	11	32
		Percent	80.8%	42.3%	61.6%
	Slight discomfort, occasional grimaces	Number	1	11	12
		Percent	3.8%	42.3%	23%
	Moderate discomfort, feet/hands tensed, tears in eyes	Number	4	3	7
		Percent	15.4%	11.5%	13.5%
	Marked discomfort apparent during the procedure	Number	0	1	1
		Percent	0%	3.8%	1.9%

**Table 3:** Patients' reports on sedation using SDFQ.

SDFQ		Local anesthetic cartridge		Total
		DEX + LIDO	LIDO alone	
I was completely relaxed during the treatment (“relaxed”)	Number	22	15	37
	Percent %	84.6	57.7	71.1
I was nervous but the treatment was carried out successfully (“slightly frightened”)	Number	3	3	6
	Percent %	11.5	11.5	11.5
I was so frightened and nervous that treatment was difficult (“severely frightened”)	Number	1	8	9
	Percent %	3.8	30.8	17.3
Total	Number	26	26	52
	Percent %	100	100	100

**Table 4:** Paired *T*-test results for VAS of patient's and surgeon's satisfaction.

	Paired differences		*t*	df	*P* value
	Mean	Sd			
PATIENT_DEX PATIENT_LIDO	0.76	1.53	2.56	25	0.017
SURGEON_DEX SURGEON_LIDO	1.46	1.77	4.20	25	<0.001
PATIENT_DEX SURGEON_DEX	−0.15	1.08	−0.72	25	0.476
PATIENT_LIDO SURGEON_LIDO	0.53	1.79	1.53	25	0.138

**Table 5:** Wilcoxon test results comparing patients' cooperation between case and control groups.

	COOPERATION_LIDO_1—COOPERATION_DEX_1	COOPERATION_LIDO_2—COOPERATION_DEX_2	COOPERATION_LIDO_3—COOPERATION_DEX_3
*Z*	−2.309^a^	−2.057^a^	−1.968^a^
Asymp. Sig. (2-tailed)	0.021	0.040	0.050

## Data Availability

Data are available from the corresponding author upon request.
